# Microbial profiles of a drinking water resource based on different 16S rRNA V regions during a heavy cyanobacterial bloom in Lake Taihu, China

**DOI:** 10.1007/s11356-017-8693-2

**Published:** 2017-03-31

**Authors:** Junyi Zhang, Congming Zhu, Rui Guan, Zhipeng Xiong, Wen Zhang, Junzhe Shi, Yi Sheng, Bingchuan Zhu, Jing Tu, Qinyu Ge, Ting Chen, Zuhong Lu

**Affiliations:** 1 State Key Lab for Bioelectronics, School of Biological Science and Medical Engineering, Southeast University, Nanjing, China; 2 Wuxi Environmental Monitoring Centre, Wuxi, China; 3 MOE Key Lab of Bioinformatics, Bioinformatics Division/Center for Synthetic and Systems Biology, TNLIST and Department of Automation, Tsinghua University, Beijing, China; 4 Wuxi Metagene Science & Technology Co., Ltd, Lake Taihu Cyanobacterial Blooms Research Institute, Wuxi, China; 5 China Environmental Protection Foundation, Beijing, China

**Keywords:** 16S rRNA, Bacterial diversity, Drinking water resources, Cyanobacterial bloom, *Microcystis*, Lake Taihu

## Abstract

Understanding of the bacterial community structure in drinking water resources helps to enhance the security of municipal water supplies. In this study, bacterial communities were surveyed in water and sediment during a heavy cyanobacterial bloom in a drinking water resource of Lake Taihu, China. A total of 325,317 high-quality sequences were obtained from different 16S ribosomal RNA (rRNA) regions (V3, V4, and V6) using the Miseq sequencing platform. A notable difference was shown between the water and sediment samples, as predominated by *Cyanobacteria*, *Proteobacteria*, and *Actinobacteria* in the water and *Proteobacteria*, *Chloroflexi*, and *Verrucomicrobia* in the sediment, respectively. The LD12 family dominated the water surface and was tightly associated with related indicators of cyanobacterial propagation, indicating involvement in the massive proliferation of cyanobacterial blooms. Alternatively, the genus *Nitrospira* dominated the sediment samples, which indicates that nitrite oxidation was very active in the sediment. Although pathogenic bacteria were not detected in a large amount, some genera such as *Mycobacterium*, *Acinetobacter*, and *Legionella* were still identified but in very low abundance. In addition, the effects of different V regions on bacterial diversity survey were evaluated. Overall, V4 and V3 were proven to be more promising V regions for bacterial diversity survey in water and sediment samples during heavy water blooms in Lake Taihu, respectively. As longer, cheaper, and faster DNA sequencing technologies become more accessible, we expect that bacterial community structures based on 16S rRNA amplicons as an indicator could be used alongside with physical and chemical indicators, to conduct comprehensive assessments for drinking water resource management.

**Electronic supplementary material:**

The online version of this article (doi:10.1007/s11356-017-8693-2) contains supplementary material, which is available to authorized users.

## Introduction

Over the past few years, the frequency and duration of cyanobacterial bloom have increased in Lake Taihu, China, despite considerable efforts to reduce nutrient pollution from the watershed (Yang et al. 2016). This is significant, because these cyanobacterial blooms negatively affect drinking water resources. Lake Taihu is a drinking water resource for more than two million people in Wuxi, China; it has been experiencing progressively more severe *Microcystis* blooms in recent decades (Chen et al. 2003). The more public attention has been drawn to this lake since the water crisis caused by a massive cyanobacterial bloom in 2007 (Qin et al. 2010). As a result, numerous studies investigated the *Microcystis* blooms and their underlying mechanisms (Ma et al. 2016; Paerl et al. 2011). However, the mechanisms underlying cyanobacterial bloom formation have not been clarified (Harke et al. 2016; Li et al. 2016). Consequently, drinking water resources in Lake Taihu remain at risk from cyanobacterial blooms. In particular, some potentially pathogenic bacteria, such as *Aeromonas*, *Vibrio*, *Acinetobacter*, and *Pseudomonas*, were detected during the cyanobacterial blooms, and these results may imply the adverse health effects on humans and animals (Berg et al. 2008).

Recent breakthroughs in microbial community profiling using 16S ribosomal RNA (rRNA) have emerged from the development of high-throughput DNA sequencing techniques, which bypasses the need for isolation or cultivation of microorganisms. High-throughput sequencing allowed for hundreds of microbial communities to be simultaneously assayed (Hamady et al. 2008). Deep sequencing of the variable region of 16S rRNA genes has become the predominant tool for studying microbial ecology. Many studies have used 16S rRNA gene amplicon sequencing to investigate bacterial communities in drinking water resources and drinking water treatment processes (Liu et al. 2014; Zeng et al. 2013). With the exception of human pathogens such as *Mycobacterium*, detected in piped water, it has been found that LD12 was detected and persisted during drinking water treatment processes (Zeng et al. 2013). However, the bacterial community, including bacterioplankton and attached bacteria in the water and sediment, has not been surveyed together using high-throughput DNA sequencing techniques in drinking water resources of Lake Taihu during a heavy cyanobacterial bloom. More importantly, bacterial community structures were introduced as a diagnostic tool for assessing watershed quality (Borruso et al. 2015). This may reveal new directions for drinking water resource management, alongside physicochemical indicators.

Therefore, the main goals of this study were (1) to profile bacterial communities in drinking water resources during a heavy cyanobacterial bloom in Lake Taihu and (2) to evaluate the effects of different V regions for surveying bacterial diversity.

## Materials and methods

### Sampling and physicochemical analyses

**Fig. 1:**
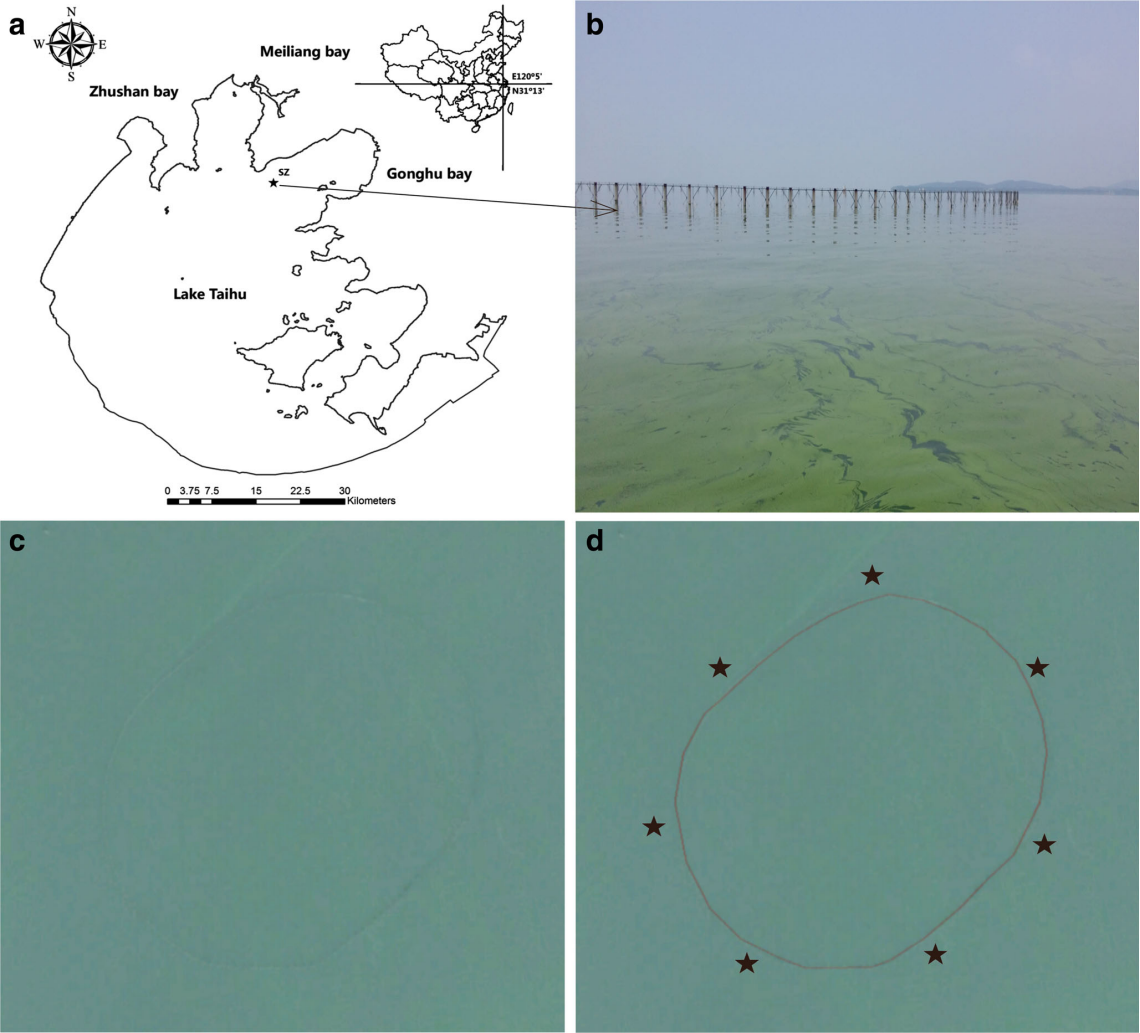
Sampling sites in Lake Taihu, China. **a** The location of SZ drinking water resources; the map was generated using ArcGIS 10.2. **b** Water intake protected by stakes. **c** The image of water intake from Google Earth. **d** Location of seven sampling sits around the water intake

The samples were collected at Shazhu (SZ) (31° 22′ 44″ N, 120° 14′ 46″ E) on August 2, 2013. SZ is the largest drinking water resource associated with Lake Taihu (Fig. 1a), supplies more than 0.6 million t/day in Wuxi, and provides more than 50% of the tap water for 6.5 million people of Wuxi. The SZ drinking water resource became famous in 2007 for a drinking water crisis (Qin et al. 2010). Therefore, our study focused on SZ. As shown in Fig. 1b–d, the water intake of drinking water resource is protected by stakes, which has an elliptical planar shape (area, 40,533 m^2^; circumference, 724 m).

Two liters of water was collected from the surface water for each sample using a 5-L Schindler sampler. Then, the seven samples were well mixed into one sample and immediately passed through a mixed cellulose ester membrane (Xingya Factory, Shanghai, China) with a 0.22-μm pore size. After filtering, the membranes containing the microorganisms were stored at −20 °C for further molecular biological analysis. The seven sediment samples were simultaneously collected with an in situ column sampler and then well mixed into one sample for further analysis. Water temperature, pH, turbidity, and dissolved oxygen (DO) were measured on location using a YSI 6600 multiparameter water quality sonde (Yellow Springs, USA). Secchi depth was measured with a white 20-cm-diameter Secchi disk lowered from the shaded side of the boat. One-liter water samples for phytoplankton identification and counts were preserved with 1% Lugol’s iodine solution (Wei and Qi 2006). The following parameters were analyzed for the water and sediment samples using standard methods (based on Chinese National Standards or Chinese industry standards): total nitrogen (TN) (HJ 636-2012), ammonium (NH_4_-N) (HJ 535-2009), nitrate (NO_3_-N) (HJ/T346-2007), nitrite (NO_2_-N) (GB/T 7493-1987), total phosphorus (TP) (GB/T11893-1989), ortho-phosphorus (PO_4_-P) (Wei and Qi 2006), chemical oxygen demand (COD_Mn_) (GB/T11892-1989), chemical oxygen demand (COD_Cr_) (GB/T 11914-1989), suspended substance (GB/T11901-1989), microcystins (GB/T 20466-2006), and chlorophyll a (Wei and Qi 2006) in the water samples. Parameters analyzed for the sediments included soil organic matter (Wei and Qi 2006), sulfide (Wei and Qi 2006), TN (Li and Li 1989), TP (Li and Li 1989), COD (Li and Li 1989), and pH (NY/T1377-2007).

### DNA extraction and amplicon generation

DNA was extracted from the filters using an E.Z.N.A. ® Water DNA Kit (OMEGA, Stamford, USA) for water samples and a Power Soil® DNA Isolation Kit (Mobio, Carlsbad, USA) for sediment samples. DNA integrity was checked by agarose gel electrophoresis and spectrophotometrically quantified in a NanoDrop ND 1000 instrument (Thermo Scientific, San Jose, USA). The V3, V4, and V6 hypervariable regions of 16S rRNA were amplified from microbial genomic DNA by PCR using barcoded fusion primers. The pool of primers is described in Table S1.

### Data processing and analysis

The V3, V4, and V6 hypervariable regions of samples were sequenced using the pair-end method with Illumina Miseq. Raw sequencing data were filtered based on the Phred scores. We took a window, which was 5 bp in size, from the first base with a 1-bp step length. The reads were trimmed if the average Phred score in the window was less than 20. We removed the processed reads that were shorter than 150 bp. After trimming, the reads were assembled using the Flash software, and the reads that could not be assembled were discarded. Then, the sequences that contained ambiguous bases, more than one mismatch in their 5′ primers, had a homopolymer greater than eight bases, or sequences length shorter than 200 bp were removed by Qiime. We also filtered the valid sequences by removing chimeras using mothur. Only high-quality sequences without chimeras were further analyzed.

We used the uclust script in Qiime to cluster high-quality sequences with a 97% similarity to obtain operational taxonomic units (OTUs). Then, the representative OTU sequences (we chose the longest sequence of each OTU as the representative sequence) were annotated by comparison to the Silva database (Release 123, https://www.arb-silva.de). The Ribosomal Database Project (RDP) classifier method in Qiime was used to infer the taxonomy classification of metagenomic samples. Additionally, OTUs were filtered using a conservative OTU threshold of *c* = 0.005% to reduce the impact of the bioinformatic analysis errors (Bokulich et al. 2013). To eliminate the effect of sequencing depth on the data analysis, each original datum was normalized using the subsample command in mothur, based on minimum sequences of the minimum sequences 40,036 and 108,439 of the sediment and water samples, respectively.

## Results and discussion

### Sample environmental parameter characterization

Sample environmental parameters are provided in Table 1. The weather during sample collection was cloudy and hot, and the water temperature reached up to 31.6 °C. *Microcystis* spp. dominated the phytoplankton assemblages, which accounted for above 98% of total cell count. The scum from *Microcystis* colonies was clearly observed at the water surface, and the DO reached 15.32 mg/L (>200% saturation), which indicates that photosynthesis was very active (Fig. 1b). Moreover, the turbidity was 91 NTU and Secchi depth was 20 cm, further indicating that this area suffered from a heavy *Microcystis* bloom.

**Table 1:** Environmental parameters of the sampling sites located in Lake Taihu, China

Variable	Water sample	Sediment sample
Total nitrogen (TN, mg/L for water, mg/kg for sediment)	1.08	789
Ammonium NH_4_-N (mg/L)	0.30	N/A
Nitrate NO_3_-N (mg/L)	0.68	N/A
Nitrite NO_2_-N (mg/L)	0.08	N/A
Total phosphorus (TP, mg/L for water, mg/kg for sediment)	0.05	1.66 × 10^3^
Ortho-phosphorus PO_4_-P (mg/L)	0.01	N/A
Chemical oxygen demand (COD, mg/L for water, mg/kg for sediment)	3.9 (COD_Mn_) 24 (COD_Cr_)	2.12 × 10^4^ (COD_Cr_)
Water temperature (°C)^a^	31.6	N/A
pH^a^	9.12	7.67
Dissolved oxygen (DO, mg/L)^a^	15.32	N/A
Turbidity (NTU)^a^	91	N/A
Secchi depth (cm)^a^	20	N/A
Suspended substance (SS, mg/L)	82	N/A
Soil organic matter (%)	N/A	1.3
Sulfide (mg/kg)	N/A	6.03
Chlorophyll a (μg/L)	145	N/A
Phytoplankton abundance (cell/L)	1.75 × 10^8^	N/A
Dominant species (percentage)	*Microcystis*(97.2%)	N/A
Toxin (MC, mg/L)^b^	0.54	N/A

*N/A* not available

^a^The results of those parameters with average of the seven samples, whereas others were assayed with a well-mixed sample

^b^The concentrations of extracellular MC-LR, MC-LR, and MC-RR were 0.31, 0.12, and 0.11 mg/L, respectively

The NO_3_-N concentration was 0.68 mg/L, highest of the different inorganic nitrogen concentrations, followed by the NH_4_-N (0.30 mg/L) and NO_2_-N (0.08 mg/L) concentrations. Typically, non-nitrogen-fixing cyanobacteria (such as *Microcystis aeruginosa*) prefer NH_4_^+^-N over NO_3_-N as an N source (Blomqvist et al. 1994), which echoed by 3.77 × 10^7^ kg N/year, which was regenerated as NH_4_^+^-N in Meiliang Bay, where the most severe *Microcystis* blooms occur (Paerl et al. 2011). In late summer, lake sediments are an N source to the water column for massive *Microcystis* bloom proliferation (McCarthy et al. 2007). The massive *Microcystis* blooms coincided with a decrease in nitrogen, which was observed by 11-year investigation in Lake Taihu during summer and autumn (Liu et al. 2011). Moreover, N limitation was proposed by nutrient loading analyses in cyanobacteria-dominated summer and fall months in Lake Taihu. The results showed N availability determined the magnitude, spatial extent, and duration of the bloom during summer-fall when the bloom potential was highest (Paerl et al. 2011). Hence, possible explanations for lower concentration of TN (1.08 mg/L), and dissolved inorganic N (DIN) dominated by NO_3_-N, mainly include higher nutrient uptake by heavy *Microcystis* bloom in the water column and nitrite oxidation across the sediment-water interface. A previous study showed that TN and TP concentrations ranged from 1000 to 1400 and 450 to 700 mg/kg, respectively, based on vertical sediment samples collected near the SZ drinking water resource from Lake Taihu in 2006 (Trolle et al. 2009). However, the surface sediment samples from drinking water resource were characterized by lower TN (789 mg/kg) and higher TP (1660 mg/kg) during heavy water blooms in this study. Since a large-scale dredging project was conducted in the SZ area during 2008, the nutrient of surface sediment should be reduced sharply; however, our results showed that TP concentration was still high. Possible explanations for higher TP concentration are mainly caused by settlement of the massive cyanobacterial blooms, whereas the loss of endogenous P is more difficult than N from the sediment (Fan and Wang 2007). Notably, the drinking water resource suffered a heavy water bloom; however, the extracellular microcystins (MCs) including MC-LR, MC-YR, and MC-RR was only 0.54 mg/L, which is lower than the limit of 1 μg/L MCs in drinking water (GB5749-2006). Nevertheless, the cellular MCs of cyanobacteria during bloom seasons should also be concerned especially as late phase of the bloom with the risk of cell degradation and death.

### Quantitative compositional sequence analysis

A total of 5544 and 8909 OTUs were obtained; these were affiliated with the 325,317 and 120,108 sequences from the water and sediment samples, respectively (Tables 2 and 3). To improve OTU credibility, we discarded OTUs using a conservative OTU threshold of *c* = 0.005%. This is a conservative threshold compared with that used by similar studies and therefore ensures high quality of the resulting data. After filtering, 320,504 and 115,938 filtered sequences were assigned to 2750 and 5938 OTUs (water and sediment, respectively) and of which 2740 and 5923 bacterial OTUs belong to 320,450 and 115,664 sequences (water and sediment, respectively) (Table S2). The species diversity and richness estimators (ACE, Chao1, Shannon, and Simpson) showed that sediment samples had higher bacterial diversity and evener distribution than water samples (Table 3). The filtered ratio of chimera removal for V6 was higher than those for V3 and V4, especially in the sediment samples (Table 2). Figure S1 shows the sequence length distribution of the V regions. V3 had the greatest standard deviation, followed by V6, and V4 had the least deviation.

**Table 2:** Statistical characteristics of V3, V4, and V6 amplicon sequences

Datasets	Water			Sediment		
	V3	V4	V6	V3	V4	V6
Subsample	108,439	108,439	108,439	40,036	40,036	40,036
Filtered sequences	105,774	107,126	107,604	37,483	39,213	39,242
Ave. sequence length (bp)^a^	158 ± 11	223 ± 6	81 ± 9	165 ± 14	220 ± 7	79 ± 8
Percentage of removing chimeras (%)	12.1	12.3	12.4	10.4	10.8	11.6

^a^The sequence length not included primers and barcodes

**Table 3:** Estimates of richness and diversity of water and sediment samples

Samples	OTUs^a^	ACE	Chao1	Shannon	Simpson	Coverage (%)
Water-V3	2318	3894	3184	4.47	0.0567	99.2
Water-V4	1446	2320	2000	5.02	0.0176	99.6
Water-V6	1780	2017	2178	6.23	0.0041	99.7
Sediment-V3	4080	5058	4972	7.09	0.0029	97.2
Sediment-V4	2280	2504	2564	6.58	0.0037	99.1
Sediment-V6	2549	2757	2867	7.06	0.0016	99.1

^a^Each of water and sediment samples included 108,439 and 40,036 sequences, respectively

### Bacterial community diversity and structure

**Fig. 2:**
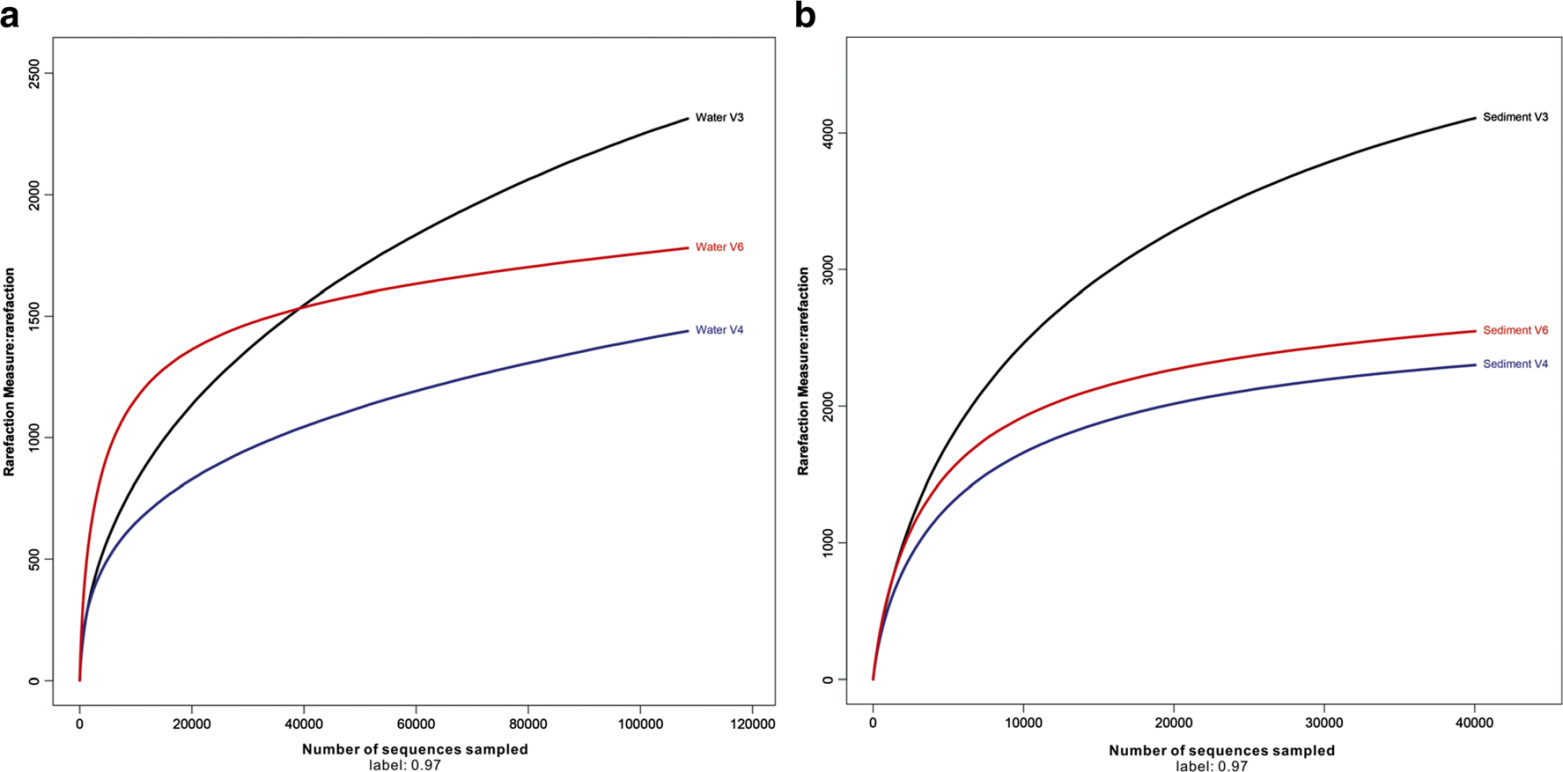
Rarefaction curves of water and sediment samples among the V regions. Curves were calculated based on OTUs at 97% similarity. **a** Water. **b** Sediment

**Fig. 3:**
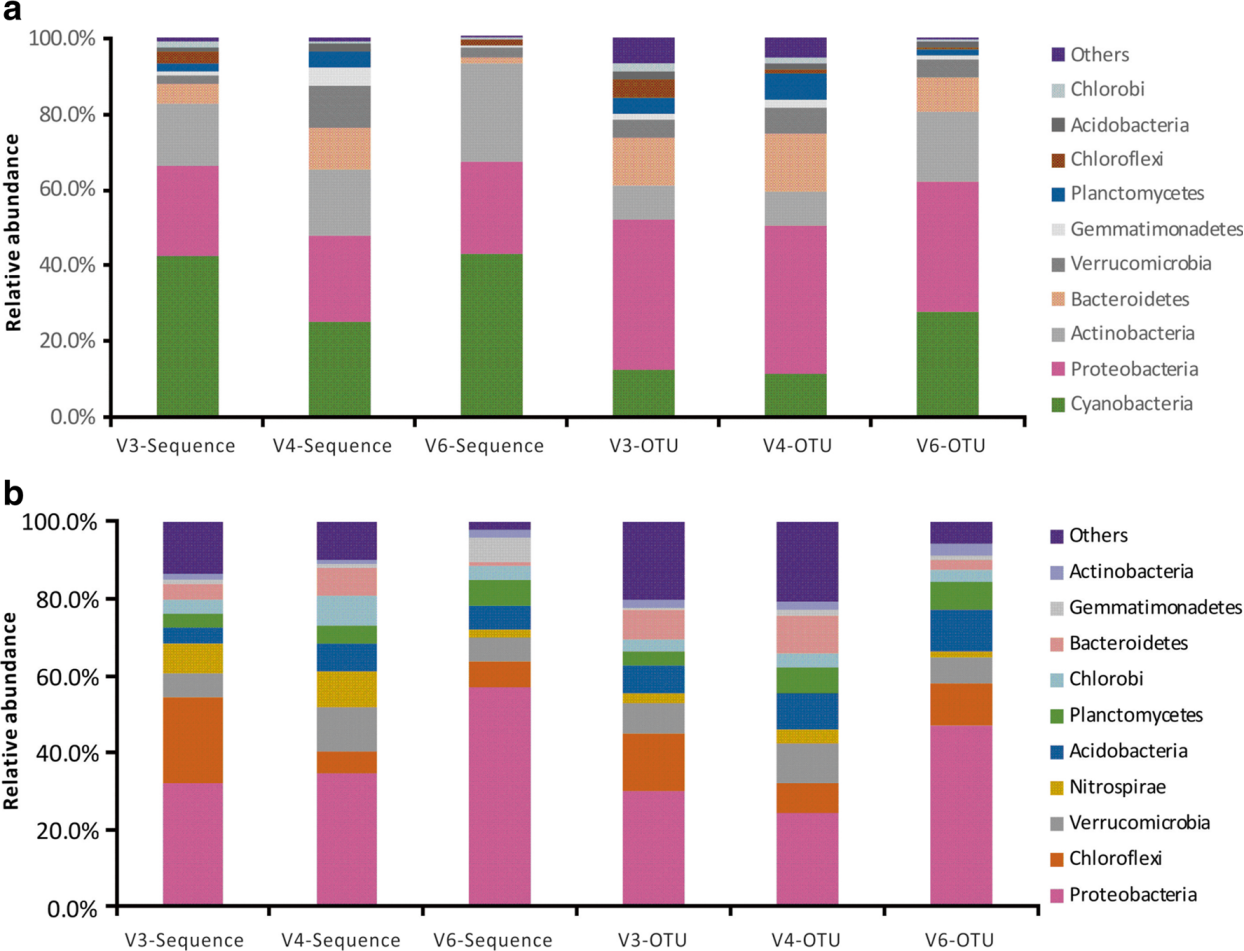
Relative abundances of bacterial taxa at the phylum level. Each *color* represents the percentage of the phylum in the total sequences and OTUs of each sample. For bacteria, only the top 10 phyla are shown. **a** Water. **b** Sediment

The combination of the rarefaction curves and high Good’s coverage indicated that this sequencing effort was sufficient to capture relatively complete diversity of these communities (Fig. 2 and Table 3). Of all filtered bacterial sequences, on average, 93.2 and 82.1% could be assigned to a known phylum; 26 and 42 phyla were detected in the water and sediment samples, respectively (Table 4). Figure 3 shows the relative abundance of sequences that were assigned at the phylum level. All 26 phyla found in the water samples were also found in the sediment samples. Although the detected phyla varied from the different V regions, *Cyanobacteria*, *Proteobacteria*, *Actinobacteria*, *Bacteroidetes*, and *Verrucomicrobia* dominated the water samples and accounted for 91.7% of total assigned sequences at phylum level (Table 5). Alternatively, the sediment samples were dominated by *Proteobacteria*, *Chloroflexi*, *Verrucomicrobia*, *Nitrospirae*, and *Acidobacteria*, which together accounted for 72.9% of total assigned sequences at the phylum level (Table 6). Obviously, *Cyanobacteria* and *Proteobacteria* were detected as the first most abundant phyla in water and sediment in this study, respectively. This is not surprising, because *Cyanobacteria* dominated the water samples as a result of this area suffering from a heavy *Microcystis* bloom.

**Table 4:** Coverage and spectrum of V regions across the taxonomic ranks in water and sediment samples

Sample	V regions	Category	Phylum	Class	Order	Family	Genus
Water	V3	Sequence^a^ (%)	91.7	90.5	82.0	76.1	51.6
		OTU^b^ (%)	76.0	70.5	54.3	43.2	22.2
		*N* ^c^	22	41	71	92	82
		Sequence^a^ (%)	98.9	98.1	91.0	85.1	56.7
	V4	OTU^b^ (%)	94.9	90.9	77.7	67.3	34.6
		*N* ^c^	24	38	75	99	99
		Sequence^a^ (%)	89.1	88.0	75.2	71.7	49.8
	V6	OTU^b^ (%)	82.2	79.5	62.9	54.5	28.8
		*N* ^c^	11	23	44	53	50
Sediment	V3	Sequence^a^ (%)	89.0	77.5	51.5	37.0	18.6
		OTU^b^ (%)	81.8	67.4	46.3	30.4	13.5
		*N* ^c^	41	71	117	138	121
	V4	Sequence^a^ (%)	89.7	81.8	55.7	38.7	21.5
		OTU^b^ (%)	88.7	73.8	53.1	37.3	17.4
		*N* ^c^	33	61	114	133	119
	V6	Sequence^a^ (%)	67.7	61.7	39.7	27.5	8.2
		OTU^b^ (%)	59.8	51.8	33.8	20.7	8.0
		*N* ^c^	20	43	84	85	64

^a^The coverage calculated with sequence

^b^The coverage calculated with OTU

^c^The annotated number for a given taxonomic path, indicating the spectrum

**Table 5:** Number of sequences, OTUs, and genera for V3, V4, and V6 for the top 10 phyla in water samples

Phylum	V3			V4			V6		
	Sequence^a^	OTU	Genus	Sequence^a^	OTU	Genus	Sequence^a^	OTU	Genus
*Cyanobacteria*	41,277 (42.6%)	73	5	26,716 (25.2%)	70	6	41,229 (43.0%)	296	5
*Proteobacteria*	23,252 (24.0%)	231	47	23,935 (22.6%)	250	57	23,574 (24.6%)	367	27
*Actinobacteria*	15,733 (16.2%)	52	2	18,554 (17.5%)	55	4	24,491(25.6%)	203	4
*Bacteroidetes*	5023 (5.2%)	77	9	11,748 (11.1%)	99	8	1909 (2.0%)	97	4
*Verrucomicrobia*	2097 (2.2%)	25	4	11,527 (10.9%)	43	5	2340 (2.4%)	47	2
*Gemmatimonadetes*	1276 (1.3%)	10	1	5259 (5.0%)	14	1	280 (0.3%)	13	1
*Planctomycetes*	1901 (2.0%)	24	4	4297 (4.1%)	44	6	420 (0.4%)	16	4
*Chloroflexi*	3180 (3.3%)	29	0	375 (0.4%)	5	1	1164 (1.2%)	9	0
*Acidobacteria*	901 (0.9%)	12	3	2055 (1.9%)	12	3	293 (0.3%)	16	2
*Chlorobi*	1725 (1.8%)	13	1	654 (0.6%)	8	1	107 (0.1%)	6	0
Others	592 (0.6%)	39	6	838 (0.8%)	33	7	7 (0.0%)	1	1

^a^The number of sequences and its percentage were presented, and the percentage was specified phylum sequences in the total of assigned sequences at phylum level (not the total of bacterial sequences). Here, sequences of V3, V4, and V6 were 96,957; 105,958; and 95,814, respectively. See Table S3 for the details. The number of sequences, OTUs, and number of genera can represent the coverage, diversity, and the genus spectrum

**Table 6:** Number of sequences, OTUs, and genera for V3, V4, and V6 for the top 10 phyla in sediment samples

Phylum	V3			V4			V6		
	Sequence^a^	OTU	Genus	Sequence ^a^	OTU	Genus	Sequence^a^	OTU	Genus
*Proteobacteria*	10,619 (32.0%)	556	48	12,221 (34.7%)	369	45	15,067 (56.8%)	550	27
*Chloroflexi*	7446 (22.4%)	274	4	1872 (5.3%)	112	3	1790 (6.7%)	132	0
*Verrucomicrobia*	1938 (5.8%)	149	4	4072 (11.6%)	158	4	1666 (6.3%)	74	1
*Nitrospirae*	2663 (8.0%)	45	1	3381 (9.6%)	57	1	569 (2.1%)	20	1
*Acidobacteria*	1403 (4.2%)	133	3	2490 (7.1%)	142	3	1637 (6.2%)	127	2
*Chlorobi*	1265 (3.8%)	63	1	2762 (7.9%)	50	1	993 (3.7%)	39	0
*Planctomycetes*	1105 (3.3%)	64	6	1549 (4.4%)	104	9	1852 (7.0%)	84	5
*Bacteroidetes*	1464 (4.4%)	140	10	2575 (7.3%)	149	8	183 (0.7%)	28	1
*Gemmatimonadetes*	256 (0.8%)	14	0	422 (1.2%)	25	0	1680 (6.3%)	16	0
*Actinobacteria*	550 (1.7%)	40	3	329 (0.9%)	33	5	510 (1.9%)	34	6
Others	4473 (13.5%)	370	41	3499 (9.9%)	313	40	595 (2.2%)	68	21

^a^The number of sequences and its percentage were presented, and percentage was specified phylum sequences in the total of assigned sequences at phylum level (not the total of bacterial sequences). Here, sequences of V3, V4, and V6 were 33,182; 35,172; and 26,542, respectively. See Table S3 for the details. The number of sequences, OTUs, and number of genera can represent the coverage, diversity, and the genus spectrum

*Proteobacteria* was the most predominant in both water and sediment; however, there was a substantial difference between the water and sediment regarding the classes of predominant *Proteobacteria*. Notably, LD12 was the dominant *Alphaproteobacteria* and contributed, on average, greater than 40% of the sequences within this class in this study’s water samples (data not shown). LD12 is a “freshwater SAR1” lineage, which was discovered in 1996 in an Arctic lake. Subsequently, it was renamed LD12 and likely originated from rare transition events of these marine SAR11 bacteria into freshwater (Pernthaler 2013). LD12 bacteria exhibited distinct population maxima in the surface layers during the summer when water temperatures exceeded 15 °C in two prealpine lakes (Salcher et al. 2013). Similar results from other studies showed that LD12 bacteria mainly thrive in the upper euphotic water layers during summer and late fall (Heinrich et al. 2013). Salcher et al. (2013) further observed that LD12 bacteria had a pronounced preference for glutamine and glutamate over seven other amino acids in situ, and they exhibited substantially higher uptake of these two substrates (and glycine) than the microbial assemblage in general. These results indicate that LD12 bacteria potentially participated in the glutamate metabolism in water, which transformed the glutamine and glutamate to amino acids that supported *Microcystis* massive proliferation. Importantly, LD12 as a bacterioplankton community was associated with high pH (Stepanauskas et al. 2003); this is consistent with our findings in this study.

The sediment samples were dominated by *Deltaproteobacteria*, identified as being a representative bacterial lineage in benthic environments. Within *Deltaproteobacteria*, the family *Desulfobacteraceae* accounted for 29.5% of total *Deltaproteobacteria* (data not shown). Most members of *Desulfobacteraceae* were known to completely oxidize organic substrates to carbon dioxide, whereas some conduct incomplete oxidation of organic substrates to acetate (Kuever 2014). Tables 5 and 6 show number of sequences, OTUs, and genera for the majority of the groups including *Cyanobacteria* and *Proteobacteria* in water and sediment.

**Fig. 4:**
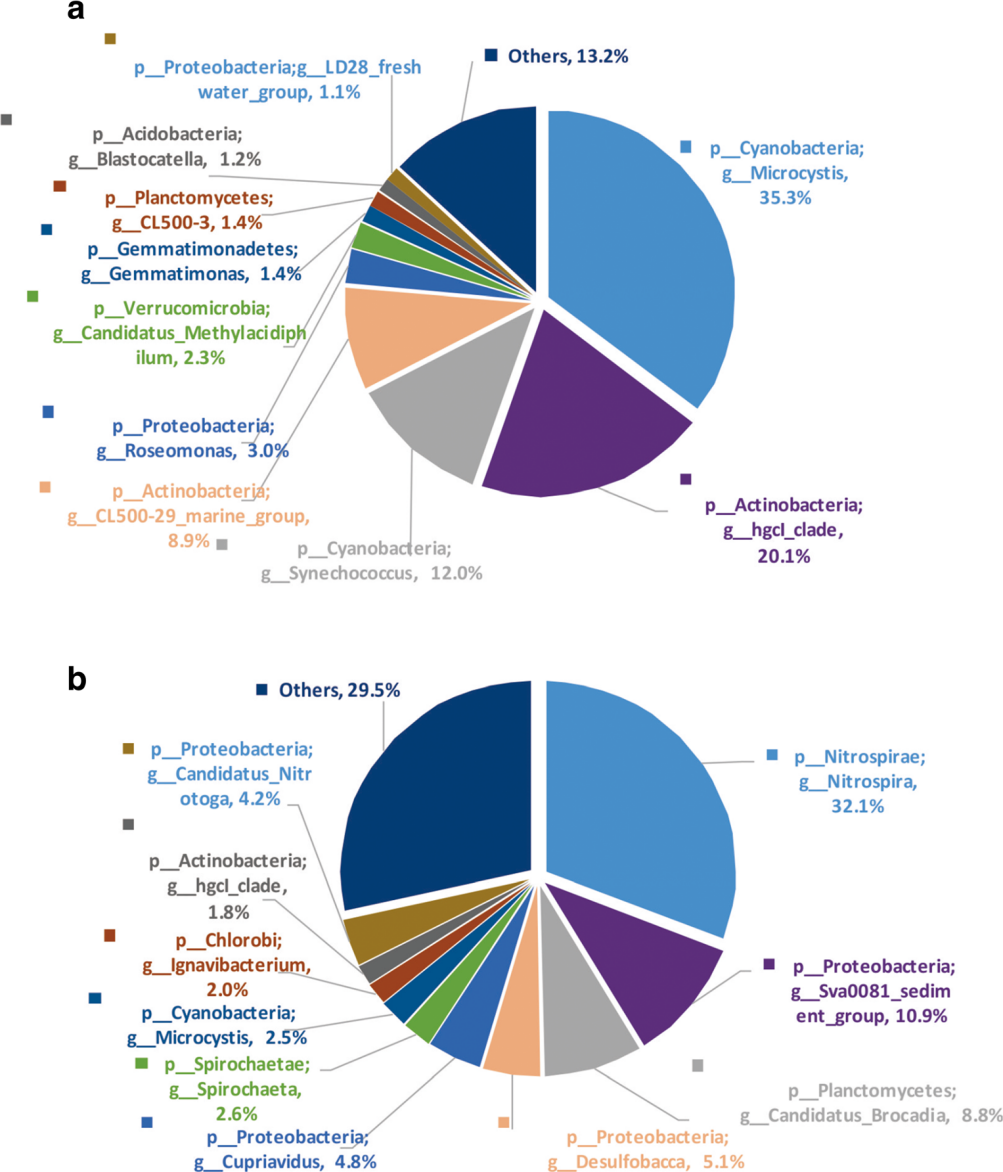
Pie chart showing the relative abundance of bacteria in water and sediment samples at the genus level. For bacteria, only the top 10 genera are shown. **a** Water. **b** Sediment

At the genus level, the most abundant genus was *Microcystis* (35.3%) in water and *Nitrospira* (32.1%) in sediment. Overall, *Microcystis*, *Nitrospira*, *hgcI_clade*, *Synechococcus*, and *CL500-29_marine_group* were the five most abundant genera (Fig. 4). Figures S2 and 3 provide more details about the abundance profiles among the V regions in the water and sediment. A total of 245 genera were obtained: 132 genera in the water and 192 genera in the sediment. Among those genera, 79 were shared between water and sediment. The most abundant bacteria at the genus level were *Microcystis* (35.3%), *hgcI_clade* (20.1%), and *Synechococcus* (12.0%), and those three genera accounted for 67.4% of total assigned sequences at the genus level in water (Fig. 4a).

Interestingly, *hgcI_clade* (affiliated with *Actinobacteria*) was the second most abundant at the genus level of the assigned sequences in water. The bacteria *hgcI_clade* was found throughout the drinking water treatment processes and accounted for 16.84% of all sequences; this indicates that it has a non-negligible role in the drinking water ecosystem (Zeng et al. 2013). This bacterium is common and abundant in a wide range of freshwater habitats, and it has a strong genetic ability to uptake carbohydrate and N-rich organic compounds (Ghylin et al. 2014). *Synechococcus* along with *hgcI_clade* was abundant in the surface water (Liu et al. 2015; Sun et al. 2014). *Microcystis* and *Synechococcus* were found to be the dominant cyanobacteria in hypertrophic water column of Lake Taihu, which were significantly correlated within the total cyanobacterial population with an *r* value that was very close to 1. Especially, *Synechococcus* dominated in Lake Taihu during the bloom season and presented a highly diverse *Synechococcus* community throughout the season. The copy number of *Synechococcus* was approximately one order of magnitude higher than that of *Microcystis* during the bloom seasons (June–September) based on the real-time PCR in water column (Ye et al. 2011). More recently, *Synechococcus* occupied a considerable percentage in the regions of Lake Taihu with low trophic levels (Cai et al. 2012).

In our previous study, *Synechococcus* was identified as present over the course of a year and thrived from April to September, with the greatest abundance in May (Li et al. 2015). Taken together, the findings indicated that there was a close relationship among *Microcystis*, *hgcI_clade*, and *Synechococcus*; all may be involved in Lake Taihu water blooms. However, those species thriving mechanism, biogeochemical cycles in aquatic systems, and mechanisms of interaction with *Microcystis* in eutrophic water need to be investigated further. *Nitrospira* was the most abundant bacteria, contributing, on average, 32.1% of the assigned sequences at the genus level in the sediment samples (Fig. 4b). *Nitrospira* was once detected in Lake Taihu sediment based on the DGGE, and six types of *Nitrospira* were observed in sediment samples (Ye et al. 2009). The large amount of *Nitrospira* detected indicated that the nitrite oxidation activity might be more active in the upper layer of the sediment.

Denitrification rates were positively correlated to NO_3_-N concentration and regulated by NO_3_-N availability in Lake Taihu (Zhong et al. 2010). Moreover, denitrification and nitrogen assimilated by *Microcystis* were the driving forces for decreasing the nitrogen content during the period of the *Microcystis* bloom in Lake Taihu (Chen et al. 2012). Even in hyper-eutrophic system, such as Lake Taihu, *Microcystis*-dominated blooms remained N-limited during the summer bloom period (Paerl et al. 2011, 2015). Hence, the higher nitrate concentration resulting from nitrite oxidation in sediment, which later enters the water column, is required to significantly proliferate *Microcystis* in the summer. This results from the relative shortage of ammonia nitrogen preferred by phytoplankton.

Importantly, pathogenic bacteria were not detected in a large amount; however, some species such as *Mycobacterium*, *Acinetobacter*, and *Legionella* were still effectively identified in very low abundances in this study. Some potentially pathogenic bacteria, such as *Aeromonas*, *Vibrio*, *Acinetobacter*, and *Pseudomonas*, living in association with cyanobacteria, were detected during the cyanobacterial blooms (Berg et al. 2008). The presence of potentially pathogenic bacteria might cause adverse human health symptoms after human contact with water that contains cyanobacteria. Therefore, it should be taken more into consideration when assessing the risks associated with cyanobacterial water blooms in drinking water resource. Moreover, some human pathogens such as *Mycobacterium* can be detected in piped water (Zeng et al. 2013). The previous studies verified that the presence of high concentrations of disinfectants was not sufficient to eliminate the survival of pathogens, such as *Legionella pneumophila* (Williams and Braun-Howland 2003; Langmark et al. 2005). An important and initial step to controlling pathogens is to develop effective monitoring strategies. Therefore, investigating microbial communities based on 16S rRNA amplicons using high-throughput DNA sequencing technologies may serve as a routine approach for monitoring water alongside physicochemical indicators to comprehensively assess drinking water resources.

### Selection of target V regions and primer sets

The metrics of “coverage” and “spectrum” were used to evaluate the performance of the V regions (Klindworth et al. 2013). Here, the coverage refers to the percentage of annotated sequences or OTUs, and the spectrum specifies the matched number for a given taxonomic path. Target V regions were compared with coverage at the domain level to assess the accuracy of bacterial capture. The number of assigned OTUs and sequences (no blast hit, Bacteria and Archaea) were compared among datasets (Table S2). For the numbers of either OTUs or sequences, V6 performed the worst for capturing bacteria. Percentages represented the relative amount of correctly captured bacterial sequences and revealed that V4 was better than V3, and V3 was better than V6. The percentage of sequences and OTUs for V4 all exceeded 99.7% after filtration, higher than those of V3 and V6. These results indicate that V4 was the most accurate for capturing the bacteria. After filtering, the percentages of the captured bacterial sequences for V3, V4, and V6 averaged 99.9% (ranging from 99.4% to 100.0%), which indicates that amplicons of variable regions were reliable and effective for surveying bacterial diversity in this study.

Table 4 shows the coverage and spectrum of different V regions across the taxonomic ranks in sediment and water samples. Table S3 provides details about the numbers of OTUs, *N* (the number of categories under specified taxonomic ranks, e.g., the number of genera that can be assigned at the genus level), and sequences. Table 4 shows that the V4 region displayed the best coverage compared with V3 and V6 across the taxonomic ranks. With regard to spectrum in the water, V4 was able to classify 24 phyla, which was better than V3 with 22 phyla and V6 with 11 phyla. With the exception of 38 classes for V4, which was lower than 41 classes for V3, the spectrum of V4 was the best across the taxonomic ranks (Table 4).

Overall, the V4 therefore yielded a better spectrum than others in the water. In the sediment, the spectrum of V3, with 41 phyla, was better than V4 (33 phyla) and V6 (20 phyla) at the phylum level. Moreover, *Synergistetes*, *SM2F11*, *Caldiserica*, *SHA-109*, *Candidate_division_TM7*, *Fusobacteria*, *Thermotogae*, *WCHB1*-*60*, and *Tenericutes* were only detected in V3, but all together only accounted for only 0.8% of total sequences assigned at the phylum level. After filtering those low populations, at a threshold of *c* = 0.005%, the spectrum of V3, with 29 phyla, was very similar to V4, with 27 phyla, and V6 only has 18 phyla. The spectrum of V3 was substantially better than those of V4 and V6 in sediment and was particularly advantageous for revealing taxa with low population densities. Therefore, the spectrum of V3 outperformed those of V4 and V6 at the phylum level in sediment. One-way-ANOVA revealed a significant difference among the V regions based on the OTUs (*p* = 0.404) and *N* (*p* = 0.224) from phylum to genus level in the water samples. This confirms that the choice of V regions is an important factor when analyzing water samples.

In addition, we found that the performance of different V regions varied widely across phyla. This finding is consistent with those of previous reports (Mao et al. 2012; Peiffer et al. 2013). Some phyla can be underrepresented or overrepresented for different V regions. For example, *TM7* was underrepresented for V3 and V5, and *Verrucomicrobia* along with *Cyanobacteria* were underrepresented for V6 (Vasileiadis et al. 2012). Furthermore, some widely used primers can miss specific phyla. For example, 784F is biased against *Verrucomicrobia*, 967F matches <5% of *Bacteroidetes*, and 1492R matches 61% of *Actinobacteria* and 54% of *Proteobacteria* and fewer than half of the other divisions (Hamady and Knight 2009). To best differentiate specific phylum, carefully selecting V regions is crucial to distinguish the bacterial diversity results based on a single 16S rRNA gene V region (Cai et al. 2013).

**Fig. 5:**
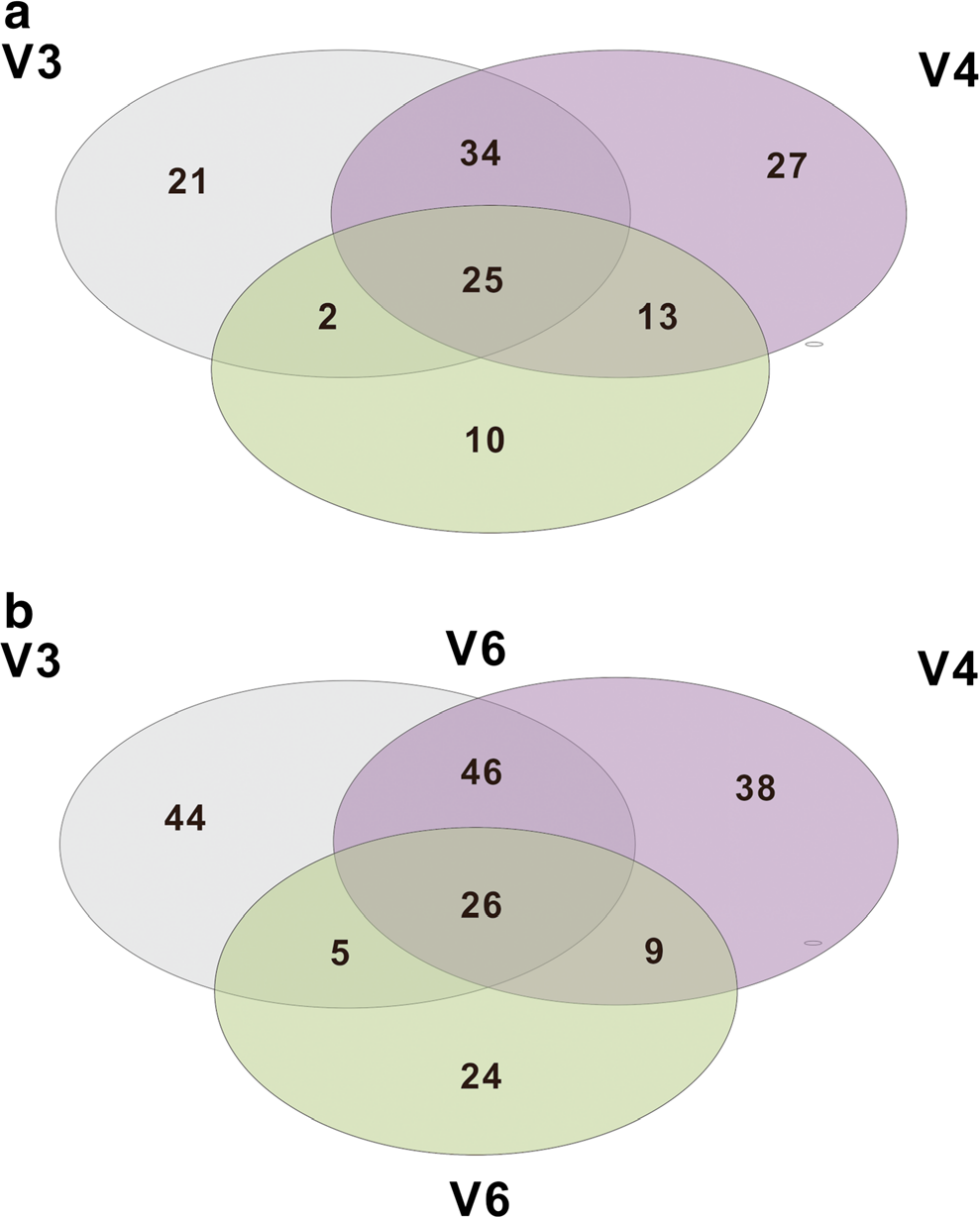
Venn diagram of the genera among the V region in the water and sediment samples. **a** Water. **b** Sediment

**Fig. 6:**
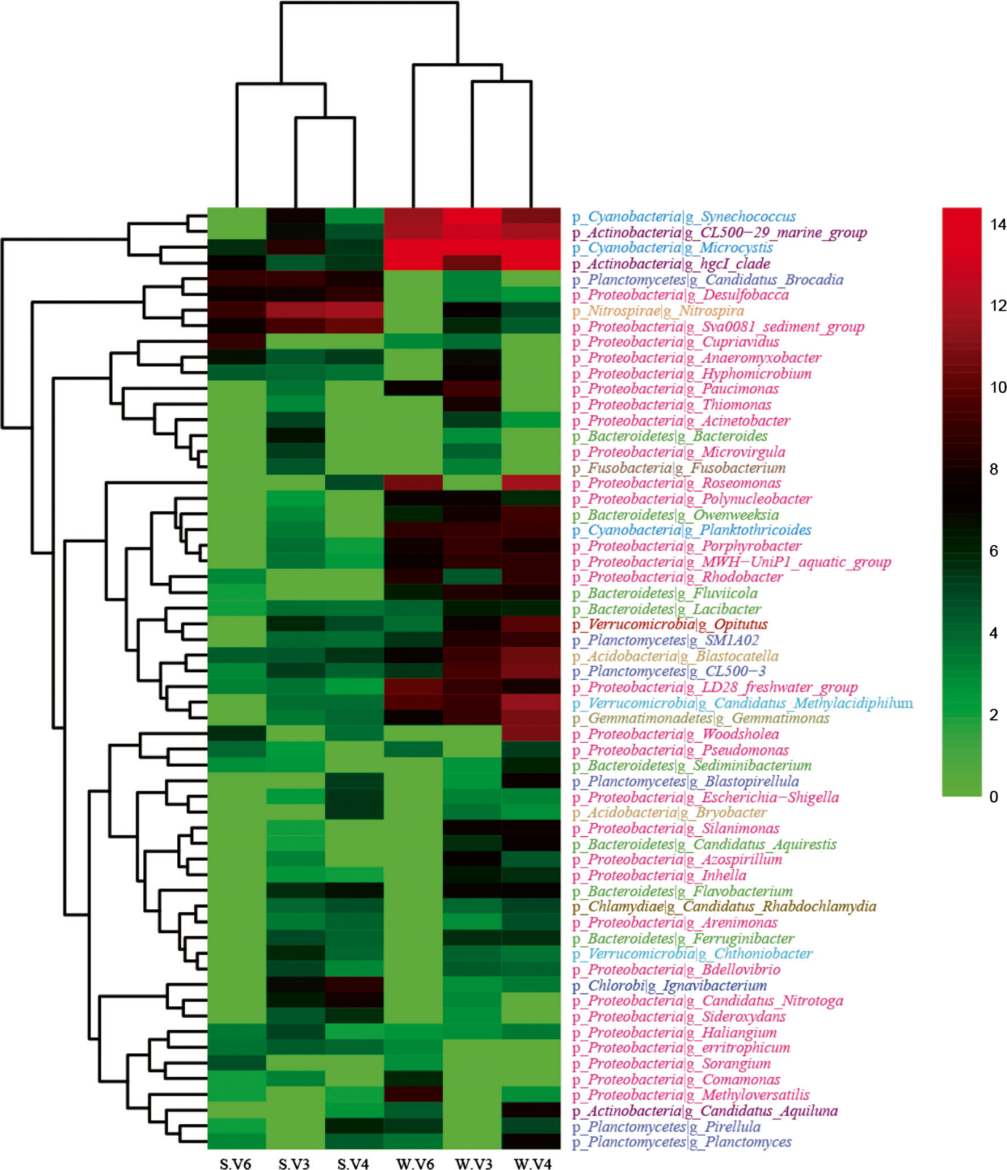
Heat map of the microbial communities based on abundance in water and sediment samples. The top 60 genera are shown, which can account for 95.5 and 80.6% of total assigned sequences at the genus level in the water and sediment, respectively

Furthermore, the genera generated from V3, V4, and V6 were compared, and Venn diagrams showed that 25 and 26 genera were shared in the water and sediment, respectively (Fig. 5). The numbers of shared genera between V4 and V3 were greater than that which either shared with V6, and the numbers of unique genera for V4 and V3 were substantially more than that of V6. The V4 estimates were closest to the estimates obtained by V3 at the genus level; this was confirmed by community composition analysis. The top 60 abundant genera were analyzed by hierarchical clustering heat map. The map showed that the abundance of most genera varied between the water and sediment samples, and the different regions can highly affect genus classification (Fig. 6). These results indicate that V4 and V3 overall outperformed V6 at the genus level in Lake Taihu.

Taken together, we suggest using V4 and V3 to investigate the bacterial diversity in water and sediment samples, respectively, based on this study for optimal coverage and spectrum from phylum to genus level (especially at the genus level). The OTU number of V6 was the highest among the different regions, which indicates that this region was more sensitive than others. However, the application of V6 was limited because of the poor coverage and spectrum. Ultimately, however, this study was primarily concerned with selection of V regions in Lake Taihu. Because this study focused on an area that suffers from heavy water blooms, the results cannot be extrapolated to other areas in Lake Taihu, especially for those regions that do not suffer from water blooms. As shown in previous studies, different community compositions severely affected the result of assessments and even yielded very different results (Youssef et al. 2009; Vasileiadis et al. 2012). Despite its preliminary nature, this study highlighted the importance of V region selection from the 16S rRNA gene-based bacterial diversity studies in Lake Taihu. Based on this study, further works are expected in the future to confirm the results. (1) Samples should include bacterial communities across various periods (one dynamic cycle) and include the different cyanobacteria bloom-forming stages. (2) Full-length 16S sequences using Sanger sequencing are necessary as a gold standard to assess the performances of the different V regions and primer sets. (3) The coverage and spectrum should be evaluated in silico with respect to the existing databases (RDP, Greengenes, and Silva) with effective evaluation of sensitivity and specificity, which can provide more useful information.

## Conclusion

This study revealed the microbial profiles of a drinking water resource in Lake Taihu (China), during a summer heavy cyanobacterial bloom. LD12 and *Nitrospira* dominated the water surface and sediment; these may be involved in the massive proliferation of cyanobacterial blooms (in water) and nitrite oxidation (in sediment). It is shown that the performance of different V regions widely varied across phyla. V4 and V3 were the most promising V regions for optimal bacterial diversity survey coverage and spectrum for water and sediment samples in Lake Taihu, respectively. Overall, the bacterial communities were effectively surveyed based on 16S rRNA amplicons in the sediment-water column, especially some pathogenic bacteria with very low abundance, and such results could make up the limitation of optical microscope observations and traditional culture-based methods. Hence, the investigation method for assessing microbial communities based on 16S rRNA amplicons could be proposed, in future, as a routine approach for water monitoring in drinking water resource management.

## Electronic supplementary material

Fig. S1 (PDF 188 kb).

Fig. S2 (PDF 160 kb).

Fig. S3 (PDF 155 kb).

Table S1 (PDF 23 kb).

Table S2 (PDF 18 kb).

Table S3 (PDF 20 kb).
