# Supplementary figures and images for: Microbial profiles of a drinking water resource based on different 16S rRNA V regions during a heavy cyanobacterial bloom in Lake Taihu, China
Source: Environ Sci Pollut Res Int. 2017 Mar 31;24(14):12796–808. doi: 10.1007/s11356-017-8693-2 (PMC5418304; doi:10.1007/s11356-017-8693-2)

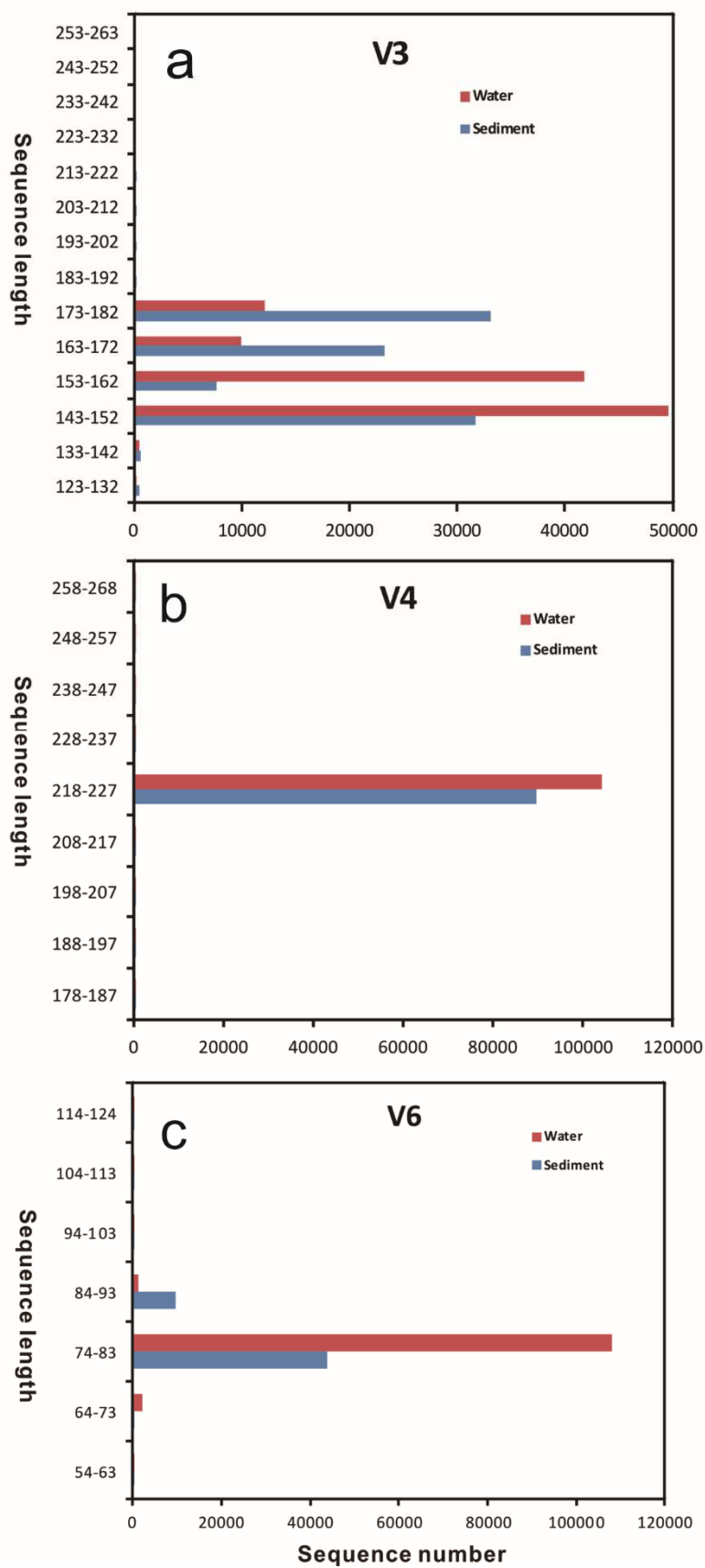

**Fig. S1** Sequence length distribution in V regions. **a** V3, **b** V4, **c** V6

Supplement: Supplementary file 1 — (PDF 188 kb) [file 11356_2017_8693_MOESM1_ESM.pdf]
