# Supplementary material for: Microbial profiles of a drinking water resource based on different 16S rRNA V regions during a heavy cyanobacterial bloom in Lake Taihu, China
Source: Environ Sci Pollut Res Int. 2017 Mar 31;24(14):12796–808. doi: 10.1007/s11356-017-8693-2 (PMC5418304; doi:10.1007/s11356-017-8693-2)
Supplement: Supplementary file 2 — (PDF 160 kb) [file 11356_2017_8693_MOESM2_ESM.pdf]

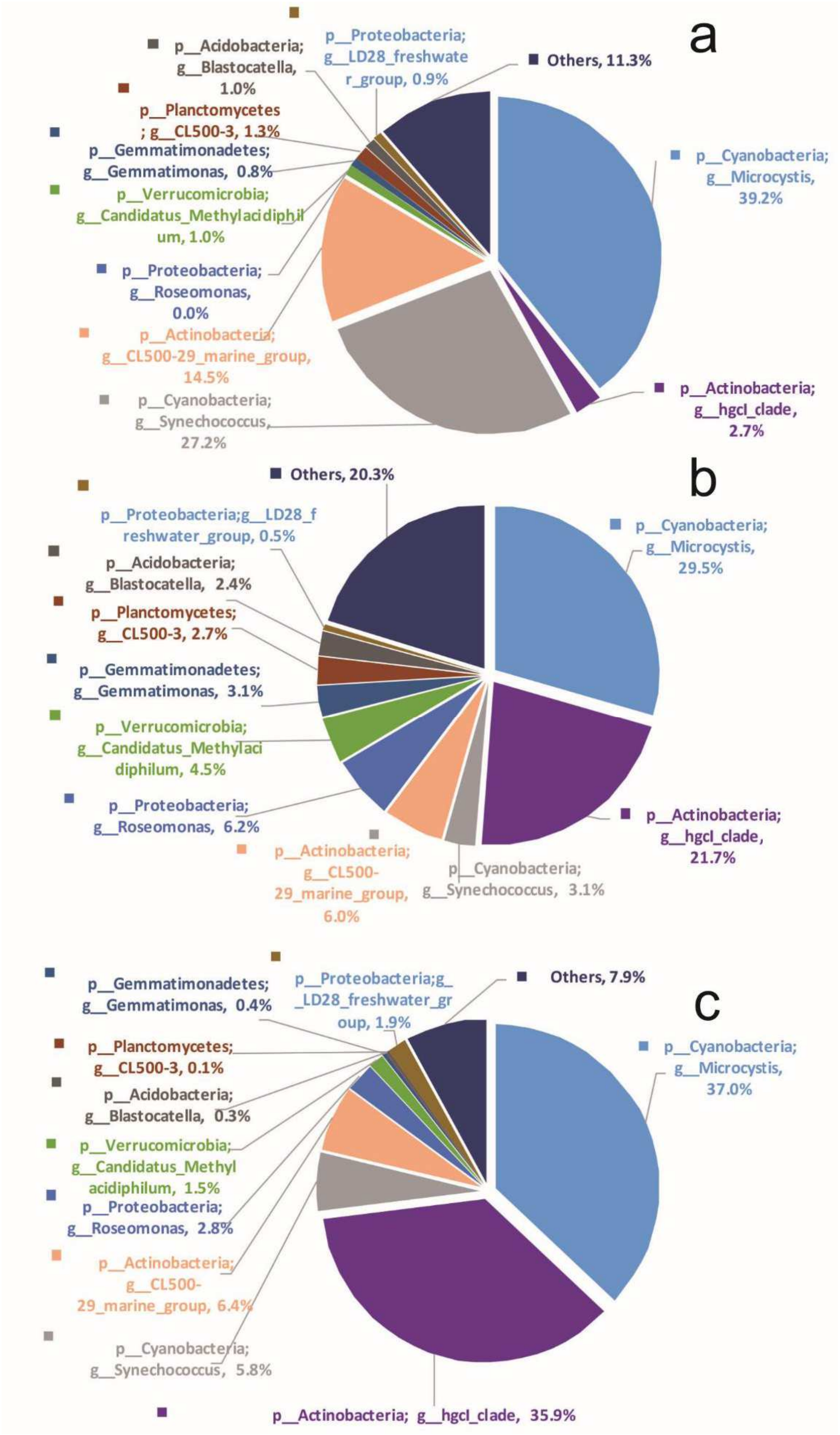

Fig. S2 Pie chart showing the relative abundance of bacteria among the V regions in water samples at the genus level. **a** V3, **b** V4, **c** V6
