# Supplementary material for: Microbial profiles of a drinking water resource based on different 16S rRNA V regions during a heavy cyanobacterial bloom in Lake Taihu, China
Source: Environ Sci Pollut Res Int. 2017 Mar 31;24(14):12796–808. doi: 10.1007/s11356-017-8693-2 (PMC5418304; doi:10.1007/s11356-017-8693-2)
Supplement: Supplementary file 4 — (PDF 23 kb) [file 11356_2017_8693_MOESM4_ESM.pdf]

Table S1 Primers used in this study

V region	Forward primer	Reverse primer
V6	986F: 5’-CNACGCGAAGAACCTTANC-4’	1046R: 5’- CGACAGCCATGCANCACCT -4’
	Bac967Fb: 5’-CAACGCGAAAAACCTTACC-4’	1046Rb: 5’- CGACAACCATGCANCACCT-4’
	Bac967Fc: 5’-CAACGCGCAGAACCTTACC-4’	1046Rc: 5’-CGACGGCCATGCANCACCT-4’
	Bac967Fd: 5’-ATACGCGARGAACCTTACC-4’	Bac1046Rd: 5’-CGACGACCATGCANCACCT-4’
	Bac967Fe: 5’-CTAACCGANGAACCTYACC-4’	
V4	520F：5’-AYTGGGYDTAAAGNG-4’	802R: 5’- TACNVGGGTATCTAATCC -4’
V3	338F：5’-ACTCCTACGGGAGGCAGCAG-4’	533R: 5’- TTACCGCGGCTGCTGGCAC-4’

The primer names are named according to probeBase 2016 (http://probebase.csb.univie.ac.at/). The best experimental conditions of PCR were exploring by the preliminary tests (temperature:45°C, 48°C, 50°C, 52°C, 55°C, 58°C, 60°C; cycles: 21, 24, 27). The results as follow: V4 and V6 amplicon samples were denatured (94°C, 5 min), followed by 27 cycles of denaturation (94°C, 30s), annealing (50°C, 30s), and extension (72°C,30s) with a final extension (72°C, 5 min). V3 amplicon samples were denatured (95°C, 5 min), followed by 25 cycles of denaturation (94°C, 30s), annealing (58°C, 30s), and extension (72°C, 30s) with a final extension (72°C, 5 min).
