# Supplementary material for: Microbial profiles of a drinking water resource based on different 16S rRNA V regions during a heavy cyanobacterial bloom in Lake Taihu, China
Source: Environ Sci Pollut Res Int. 2017 Mar 31;24(14):12796–808. doi: 10.1007/s11356-017-8693-2 (PMC5418304; doi:10.1007/s11356-017-8693-2)
Supplement: Supplementary file 5 — (PDF 18 kb) [file 11356_2017_8693_MOESM5_ESM.pdf]

Table S2 OTU and sequence results for V3, V4 and V6 from sediment and water samples

V region	Category	Raw (sediment)		Filtered (sediment)[a]		Raw (water)		Filtered (water)[a]	
		OTU	Sequence	OTU	Sequence	OTU	Sequence	OTU	Sequence
V3	No blast hit	12	466	2	178	6	621	1	6
	Archaea	56	155	1	31	33	324	2	23
	Bacteria	4,012	39,415	2,259	37,274	2,279	107,494	770	105,745
V4	No blast hit	12	53	0	0	2	44	0	0
	Archaea	7	31	1	1	6	132	2	5
	Bacteria	2,061	39,952	1,705	39,212	1,438	108,263	667	107,121
V6	No blast hit	83	1,045	6	45	21	1,125	4	15
	Archaea	23	345	5	19	33	240	1	5
	Bacteria	2,295	38,646	1,959	39,178	1,654	107,074	1,303	107,584

[a] OTUs were filtered using a conservative OTU threshold of c = 0.005%.
