# Supplementary material for: Microbial profiles of a drinking water resource based on different 16S rRNA V regions during a heavy cyanobacterial bloom in Lake Taihu, China
Source: Environ Sci Pollut Res Int. 2017 Mar 31;24(14):12796–808. doi: 10.1007/s11356-017-8693-2 (PMC5418304; doi:10.1007/s11356-017-8693-2)
Supplement: Supplementary file 6 — (PDF 20 kb) [file 11356_2017_8693_MOESM6_ESM.pdf]

Table S3 OTU, N and sequence results among different V regions across taxonomic ranks in sediment and water samples

V region	Simples	Category	Phylum	Class	Order	Family	Genus
Water	V3	Sequence	96,957	95,653	86,695	80,439	54,583
		OTU	585	543	418	333	171
		N[a]	22	**41**	71	92	82
	V4	Sequence	**105,958**	**105,135**	**97,501**	**91,139**	**60,710**
		OTU	633	606	518	449	231
		N[a]	**24**	38	**75**	**99**	**99**
	V6	Sequence	95,814	94,674	80,880	77,090	53,562
		OTU	**1,071**	**1,036**	**819**	**710**	**375**
		N[a]	11	23	44	53	50
Sediment	V3	Sequence	33,182	28,870	19,192	13,804	6,935
		OTU	**1,848**	**1,522**	**1,046**	**686**	**306**
		N[a]	**41**	**71**	**117**	**138**	**121**
	V4	Sequence	**35,172**	**32,073**	**21,842**	**15,193**	**8,448**
		OTU	1,512	1,258	906	636	297
		N[a]	33	61	114	133	119
	V6	Sequence	26,542	24,161	15,562	10,766	3,202
		OTU	1,172	1,014	662	406	156
		N[a]	20	43	84	85	64

[a] The number of categories under specified taxonomic ranks. e.g., Number of the genera at the genus level. The maximum (OTU, N, and Sequence) among the different regions marked in bold.
